# Ultra-Scaled Si Nanowire Biosensors for Single DNA Molecule Detection †

**DOI:** 10.3390/s23125405

**Published:** 2023-06-07

**Authors:** Aryan Afzalian, Denis Flandre

**Affiliations:** 1 Imec, 3001 Leuven, Belgium; 2 UCLouvain, 1348 Louvain-la-Neuve, Belgium

**Keywords:** biomolecule, DNA, ions, MOSFETs, nanotechnology, quantum wires, semiconductor device modeling

## Abstract

In this study, we use NEGF quantum transport simulations to study the fundamental detection limit of ultra-scaled Si nanowire FET (NWT) biosensors. A N-doped NWT is found to be more sensitive for negatively charged analytes as explained by the nature of the detection mechanism. Our results predict threshold voltage shifts due to a single-charge analyte of tens to hundreds of mV in air or low-ionic solutions. However, with typical ionic solutions and SAM conditions, the sensitivity rapidly drops to the mV/q range. Our results are then extended to the detection of a single 20-base-long DNA molecule in solution. The impact of front- and/or back-gate biasing on the sensitivity and limit of detection is studied and a signal-to-noise ratio of 10 is predicted. Opportunities and challenges to reach down to single-analyte detection in such systems are also discussed, including the ionic and oxide-solution interface-charge screening and ways to recover unscreened sensitivities.

## 1. Introduction

Nanoscale devices, such as Si nanowire FETs (NWTs) [1,2,3,4,5,6,7,8,9,10,11] or graphene nanoribbon and carbon nanotube FETs [12,13,14,15,16], are promising transducers for the label-free detection of biological species. Both theoretical and experimental studies have predicted improvement of sensitivity (S) when scaling down NWTs dimensions. In-depth studies of S and physics of Si nanowire transducers scaled in the nanoscale regime are, however, lacking. Semi-classical models (e.g., Drift–Diffusion), which do not capture quantum effects and use macroscopic mobility models, are typically used for simulating transport in such systems [4]. Here, we investigate the physics and properties of deeply scaled Si NWT biosensors (NWTBs) using an in-house quantum-transport simulator based on the Non-Equilibrium Green’s Functions (NEGF) formalism [17,18], which we augmented to include the simulation of ions and analytes in solution. In particular, we investigate the opportunities and challenges to achieve single-analyte detection in such a system, where S is expected to be the highest and quantum effects are strong [19]. We focus here on the transduction part, assuming that the analyte can reach the device. In a real system, an array of such ultra-sensitive detectors should be used to achieve both a high probability of detection and an overall single-analyte resolution range [20].

In Section 2, the nanowire device structure under study and the simulation models are discussed. Section 3 presents our results. Section 3.1 investigates the fundamental physics and sensitivity of ultra-scaled NWTs in the presence of a single charge. The impact of charge polarity, cross-section size, and channel length are investigated in both air and solution for various ionic contents. The demonstration and understanding of the physical phenomena responsible for the asymmetric response of an NWT versus charge polarity and the different decrease rates for each polarity of S versus channel length are also presented. The important effect of pH-dependent oxide interface charges and the role of passivation by the surface functionalization on S are quantified as well. Leveraging on the results of the previous section, Section 3.2 then investigates the sensitivity and limit of detection of the concrete case of an optimized NWT for detecting a 20-base-long DNA molecule. The opportunities and challenges for reaching single-analyte detection in such a system are then quantified for physiological-like (100 mM) and dilute (1 mM) ionic solutions. The impact of front- and back-gate biasing, as well as bottom oxide (BOX) thickness as a mean to increase the sensitivity and limit of detection, are also investigated and discussed.

## 2. Materials and Methods

**Figure 1:**
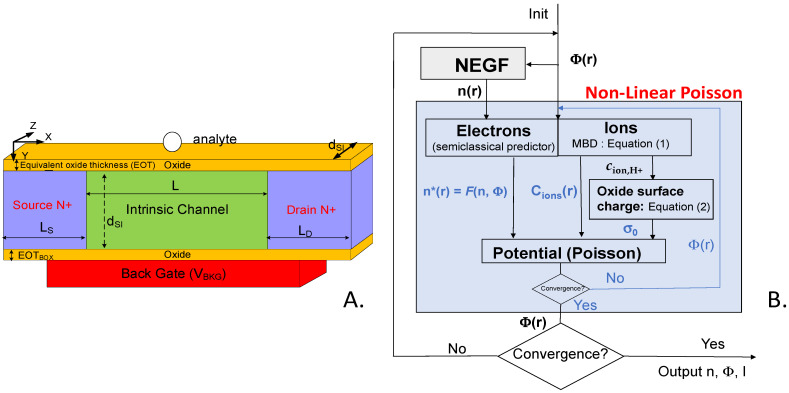
(**A**) Schematic view of the studied [100] square-cross-section N-type nanowire. The top and lateral gates (not shown on the figure) are left floating. (**B**) Self-consistent simulation flowchart used to simulate the Si NWTBs in solution.

**Figure 2:**
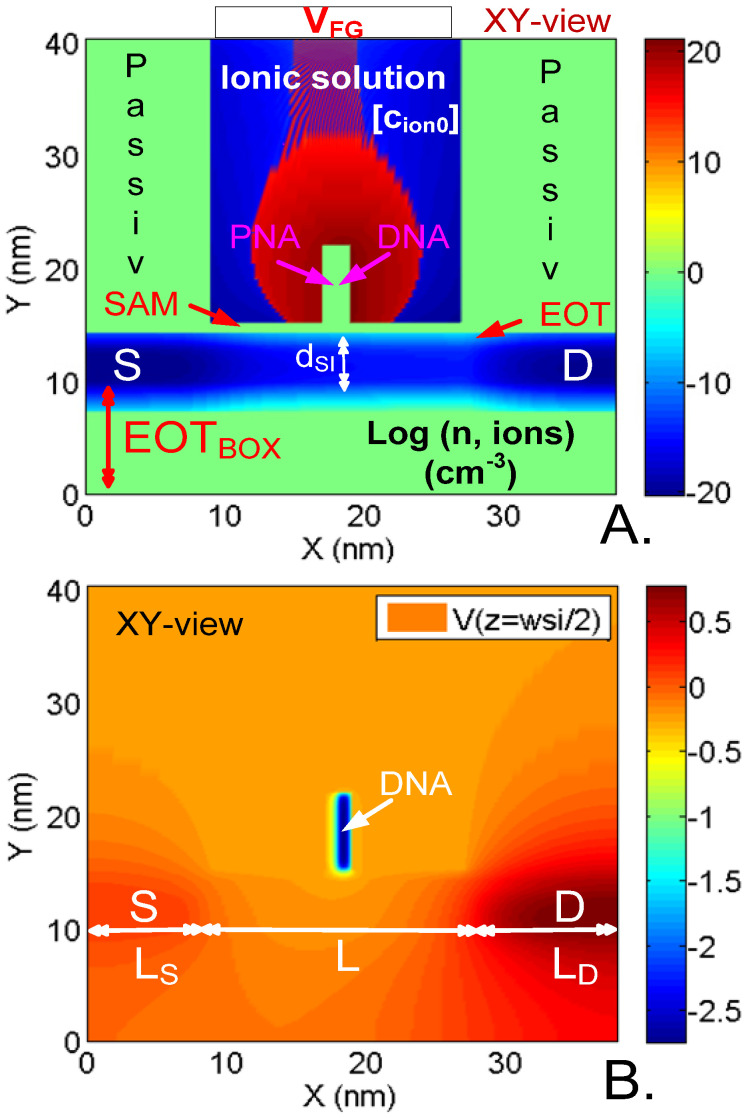
Surface plots in the Z-plane cutting across the middle of the NW of the simulated (**A**) carrier concentration (electrons, ions), and (**B**) potential profile. The NW has a 20-base-long DNA single strand (with 1 negative charge/base) linked to its complementary PNA (0 charge/base) receptor on the top gate/SAM linker stack and a 25 nm surrounding 100 mM NaCl ionic solution. d_SI_ = 3 nm, L = 20 nm. S/D extensions: L_S_ = 8 nm L_D_ = 12 nm, V_D_ = 0.7 V; doping N_+_ = 10^20^ cm^−3^. Gates: V_G_ = −0.25 V. V_BKG_ is left floating. Oxide: Front: Al_2_O_3_, EOT = 0.5 nm. Back: SiO_2_, EOT_BOX_ = 10 nm. SAM layer of thickness t_SAM_ = 1 nm, permittivity ε_R,SAM_ = 2.3. T = 300 K. Reprinted with permission from Ref. [21]. 2014, John Wiley & Sons.

The device under study is a N-doped NWT with a square cross-section of side-dimension d_SI_. The channel is intrinsic (Figure 1A). The back-gate oxide (BOX) has an equivalent thickness EOT_BOX_. The side and top gates are floating and consist of an oxide layer of equivalent thickness EOT. In order to increase the selectivity of the NWT, dielectric self-assembled monolayers (SAM) can also be added on top of these gates. The SAM affinity-based layers are assumed to be flat and homogeneous with a thickness t_SAM_ and a relative dielectric permittivity *ε*_rSAM_. A charged analyte with charge *z*_a_.*q* is placed on the top gate, centered at half the channel length and width. On the NWT top and sides, a surrounding media is considered and taken wide enough so that the results do not change when further increasing its thickness (typically from 10 to 100 nanometers). Both air and a NaCl solution with an ionic concentration *c*_ion0_ were considered (Figure 2). The NWT can be either biased by its back gate (V_BKG_) located under the channel below the BOX (Figure 1A), or, when in solution, biased by a fluid gate (V_FG_) assumed in Faradaic contact with the solution (Figure 2).

To model the ions in solution, we assume that each species *i* with charge valence *z*_i_ follows a modified Boltzmann distribution (MBD). The MBD is a Boltzmann distribution modified to include, using an effective ion diameter *d_i_*, steric effects that become important at large voltage (Φ(*r*)) [22]:

(1) $$c_{ion,i}(r)=z_{i}.c_{ion0,i}\frac{e^{\frac{z_{i}\Phi (r)}{2U_{t}}}}{1+2\nu_{i}\;\sinh^{2}(\frac{z_{i}\Phi (r)}{2U_{t}})}{}_{}\;{{with}^{}}^{}\;\nu_{i}=2d_{i}^{3}.c_{ion0,i}$$

In Equation (1), *U_t_* is the thermal voltage, while *v_i_* is the adimensional packing parameter related to *d_i_* (*d_Na_* = 3.68 Å, *d_Cl_* = 2.42 Å) [23]. Although usually in negligible concentration when compared to other ions in buffered solutions, considering the natural water ions, and in particular *H*^+^, is of importance in order to account for the pH and voltage-dependent surface charge density related to the chemical reaction at the oxide–solution interface (*r* = (*x*,*y*,*z*) = *r_ox-sol_*) between the local H^+^ ions and the oxide [24]. For computing the *H*^+^ concentration, Equation (1) can be used with *c_ion_*_0,*H+*_ *=* 10^3^ × *N*_avo_ *×* 10*^−pH^ *m^−3^, where *N*_avo_ is the Avogadro number. From there, the surface charge density (*σ*_0_) can be computed using the site-binding model [25]:

(2) $$\sigma_{0}=q.N_{S}\frac{a_{H_{S}^{+}}^{2}-K_{a}K_{b}}{K_{a}K_{b}+K_{b}a_{H_{S}^{+}}^{}+a_{H_{S}^{+}}^{2}}{}_{}\;and^{}\;{}^{}a_{H_{S}^{+}}^{}={\frac{c_{ion,H^{+}}(r=r_{ox-sol})}{10^{3}.N_{avo}}}^{}(\mathrm{mole}/l)$$

where $a_{H_{S}^{+}}^{}$ is the activity or Molar concentration of the interface (*r = r_ox-sol_*) *H*^+^ ions. The dimensionless intrinsic dissociation constants *K_a_* and *K_b_*, as well as the density of available sites, *N_S_*, are oxide-dependent [24].

To simulate the electron transport in the nanowires, we used our Self-Consistent NEGF simulator [17,18] that achieves good agreement with other NEGF simulators [26,27] and experimental results [28]. The electronic wave functions were calculated in the Si and surrounding oxides. We used an effective mass (EM) Hamiltonian including NWT diameter-dependent effective masses and non-parabolic corrections that were extracted from a sp^3^d^5^s* Tight-Binding Hamiltonian [17]. Phonon scattering was included using the Self-Consistent Born approximation [18]. The real space (RS) equations for retarded (*G^R^*), lesser (*G*^<^) and greater (*G*^>^) Green’s functions read [29]:

(3) $$G^{R}={\left(EI_{N}-H-\Sigma^{R}\right)}^{-1}$$

(4) $$G^{<}=G^{R}\Sigma^{<}G^{R†}$$

(5) $$G^{>}=G^{R}-G^{R†}+G^{<}$$

where *E* is the scalar energy, *I*_N_ the identity matrix, *H* the device Hamiltonian and Σ*^R^*^,<^ the retarded, lesser self-energies that include the interaction terms (e.g., with the semi-infinite leads Σ*_C_^R,^*^<^ and the electron–phonon scattering terms Σ*_S_^R^*^,<^) are matrices of rank *N*, i.e., the number of degrees of freedom (typically for EM the pseudo-atomic positions of a cubic-atomic lattice with mesh parameter *a* = 0.25 nm).

From the Green’s functions, the NEGF-computed RS electron density, *n*, at position *r_i_*, and the device current *I_kl_* from any cross-section slab *k* to its next neighboring slab *l*, can be computed by:

(6) $$n\left(r_{i}\right)=\frac{n_{s}n_{v}}{2\pi }\int_{-\infty }^{\infty }-iG<\left(r_{i},r_{i},E\right)dE$$

(7) $$I_{kl}=\frac{n_{s}n_{v}}{2\pi }\frac{q}{n}\int_{-\infty }^{\infty }trace\left(H_{kl}G_{ik}^{<}-G_{kl}^{<}H_{lk}\right)dE$$

where *n*_S_ (*n_V_*) is the spin (respectively, the valley) degeneracy factor. We note that we did not directly solve the RS equations. We instead performed a unitary transformation of the NEGF matrices using a coupled-mode-space (CMS) approach [17,18]. The CMS NEGF method is faster than the real-space algorithm, while it conserves the required accuracy by preserving the mode coupling in the vicinity of the perturbation potential induced by the charged analytes [17,30].

Finally, as depicted in the simulation flowchart of Figure 1B, the charge distributions (electrons and ions, as well as *σ*_0_) were included to compute the potential in our finite-difference-method (FDM)-based non-linear-Poisson (NLP) solver. To stabilize and expedite the self-consistent convergence, we predict in an inner loop of our NLP solver the carrier changes with respect to potential variations (Figure 1B). For electrons, the NEGF electron density is first interpolated to the Poisson mesh using a tri-linear interpolation method. Semi-classical predictors based on Fermi–Dirac integrals are then used to estimate the NEGF density changes due to potential variations [27]. The ions and surface charges are directly solved on the Poisson FDM mesh using Equations (1) and (2). After the potential convergence in the NLP solver is achieved, a tri-linear interpolation is used to interpolate the 3D-potential Φ(*r*) onto the NEGF-solver cubic lattice to update the device Hamiltonian and a new outer iteration is started. The procedure is self-consistently iterated until the charge and current variations in the outer loop are below 1%. Dirichlet boundary conditions were used for the gate electrodes, while Neumann boundary conditions were used elsewhere.

**Figure 3:**
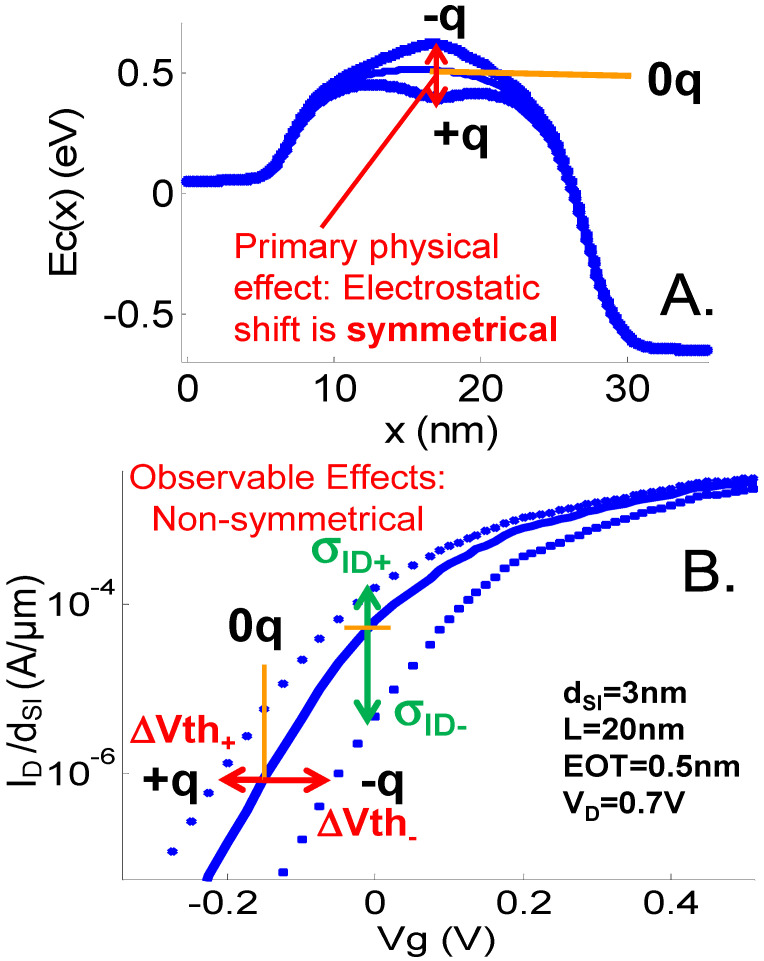
(**A**) Conduction band minimum E_c_(x) along the channel and (**B**) simulated I_D_(V_G_) curves of a d_SI_ = 3 nm NWT with L = 20 nm and with an analyte with *z*_a_ = −1, 0, and 1 in air. V_D_ = 0.7 V, EOT = EOT_BOX_ = 0.5 nm. t_SAM_ = 0 nm. The analyte charge is distributed in a volume of 0.25 × 0.25 × 0.25 nm^3^. The primary physical effect, which is an electrostatic shift on the energy sub-bands (**A**), results in a shift in the I_D_(V_G_) characteristics (**B**).

**Figure 4:**
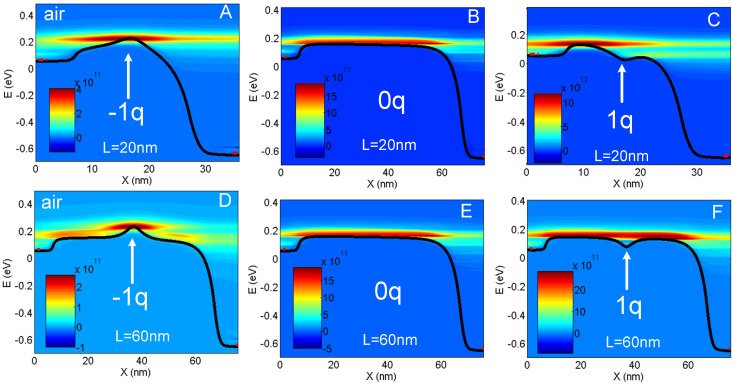
Current spectrum J(x,E) in A/J (surface plot) along the channel (x) direction for the L = 20 nm and L = 60 nm NWTs in air with *z*_a_ = −1 (**A**,**D**), *z*_a_ = 0 (**B**,**E**) and *z*_a_ = 1 (**C**,**F**, respectively). The conduction band E_c_(x) is also plotted (black line). The position of the S and D Fermi levels is indicated by a red dot at the S and D sides. d_SI_ = 3 nm. Originally published in [19].

The uses of the NEGF allows for a rigorous treatment of quantum effects such as confinement and tunneling that play a significant role on nanowires with nanometer-size diameters and a few tens-of-nanometers channel lengths. Furthermore, in such a formalism, the influence of the analyte and possible ions is directly included in a non-perturbative and exact way through the electrostatic potential that is solved for in the full domain, NWT and surrounding media (Figure 2 and Figure 3). This allows for naturally taking into account the microscopic transport changes induced by the analyte (Figure 4). Such changes results in a change of the device macroscopic mobility and impact both current and sensitivity. In the case of Figure 4D, for instance, one can observe that due to the negatively charged analyte, the microscopic transport is different from the case without an analyte (Figure 4E). The band bending induced by the presence of the analyte triggers inelastic electron–phonon scattering. This, in turn, strongly reduces the velocity overshoot that is present in a 60 nm long device without an analyte.

## 3. Results

### 3.1. Physics and Sensitivity in the Presence of a Single Charge

In this section, we introduce various definitions of the NWTB sensitivity and summarize, for completeness, the fundamental trends of their detection limit, when using as analyte a simple electronic charge (i.e., the smallest detectable charge unit) [18]. To compare air and solution results, we shall assume that the NWT is only biased by its back gate (V_G_ = V_BKG_). We furthermore assume that EOT = EOT_BOX_ = 0.5 nm (Figure 2). This leads to very similar sensitivities to those achieved by the commonly used front-gate-biasing scheme (V_G_ = V_FG_). The exact impact of these assumptions will be discussed later in this paper.

#### 3.1.1. Sensitivity: Physics and Definitions

When in the presence of a positively (+q case) or negatively (−q case) charged analyte, the energy sub-bands and, hence, the I_D_(V_G_) characteristics are shifted in opposite directions (Figure 3). The sensitivity can be estimated in mV/|q|, both for a positive (*z*_a_ = 1) and negative (*z*_a_ = −1) charge, i.e., respectively, S_+_ and S_−_, as the gate voltage shift needs to achieve the same current in the characteristics for both charged and uncharged (*z*_a_ = 0, 0q case) cases at a given reference current, I_D,0q_, or equivalently V_G,0q_ (I_D_ or V_G_ of the uncharged case) (Figure 3). This gate voltage shift is often abusively called the threshold voltage shift, ΔV_TH_, although it is in fact bias-dependent (the I_D_(V_G_) characteristics are not just shifted horizontally). Indeed, ΔV_TH_ typically decreases for increasing NWT inversion levels (ΔV_TH_ is a decreasing function of I_D_ or V_G_). The reason is that the I_D_(V_G_) characteristics slope is modified by the presence of the charged analyte. For the −q (+q) case, the slope is improved (degraded) as the negative charge pushes the channel closer to the back gate (closer to the front gate), and hence improves (degrades) the gate coupling. The variation of ΔV_TH_ with the back-gate bias is further amplified when using a fluid-gate biasing scheme, as will be discussed at the end of this paper.

It is also possible to measure the current variation, Δ*I_D_*, (vertical shift) at a fixed gate voltage (Figure 3). This leads to a current-based sensitivity definition:

(8) $$\sigma_{I_{D}}(V_{G})=\frac{\Delta I_{D}(V_{G})}{\min(I_{D,i}(V_{G}))}$$

The subscript *i* in *I_D,i_* stands for the minimum of the charged or uncharged current that is used for the normalization. This is used to achieve consistent results when comparing +q and −q cases. As Δ*V_TH_* and $\sigma_{I_{D}}$ are related through the transistor current–voltage characteristics, the best current sensitivity is achieved in a subthreshold regime, where the relation is exponential [18].

#### 3.1.2. Impact of Physical Parameters on the Unscreened Sensitivity

The impact of the cross-section diameter on the sensitivity of a 20 nm long N-NWT in air has been studied in [18]. In all cases, the sensitivity to a single charge is as high as several tens to hundreds of mV, translating to order-of-magnitude changes in current levels in the subthreshold regime. The general trend, however, is the reduction of the NWT sensitivity when increasing d_SI_, as could be expected from the nature of the electrostatic interaction which reduces with distance. Interestingly, a dissymmetry can be observed: |S_−_| is larger than S_+_ (Figure 3B) and the dissymmetry rapidly increases with L [18].

A negative charge locally creates a barrier in the conduction band and increases both the top of the channel barrier (TCB) and V_TH_. A positive charge results in a local potential well by lowering the electron energy. As a result, TCB and V_TH_ decrease (Figure 3A and Figure 4). The height of the barrier or the depth of the well increase for smaller cross-sections. In the −q case, S_−_ is directly linked to the charge-induced barrier. For longer channels, the enhanced inelastic scattering allows for more electrons with an energy lower than TCB to overcome the barrier by absorbing phonons. Hence, the sensitivity decreases with L, but rather slowly (Figure 4A,D).

In the +q case, S_+_ and the V_TH_ shift are not directly induced by the potential well created by the charge, but rather by its efficiency to lower TCB. S is therefore lower than in the barrier case and rapidly decreases as L is increased (Figure 4C,F). Additionally, as S_+_ is less sensitive to the direct analyte electrostatic interaction (i.e., the depth of the potential well), secondary effects, e.g., short channel effects, may play a more significant role on S_+_ value. We hence observed a reduced S_+_ dependency on d_SI_ when compared to S_−_ [18].

#### 3.1.3. Ions and Surface Charge Screening in Solution

**Figure 5:**
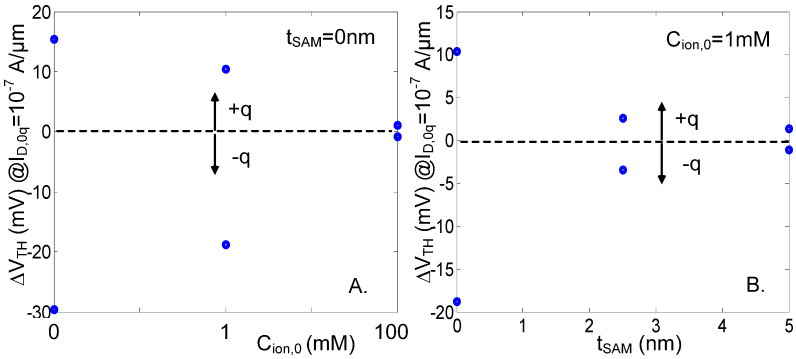
Simulated V_TH_ shift compared to the 0q case for a charged analyte with *z*_a_ = −1 and +1, extracted at I_D,0q_ = 10^−7^ A/µm in solution with no oxide charges and d_SI_ = 2 nm (**A**), in function of c_ion0_ with t_SAM_ = 0 nm, (**B**) in function of t_SAM_ with c_ion0_ = 1 mM and ε_rSAM_ = 2.3. V_D_ = 0.7 V. EOT = 0.5 nm. L = 20 nm. Originally published in [19].

**Figure 6:**
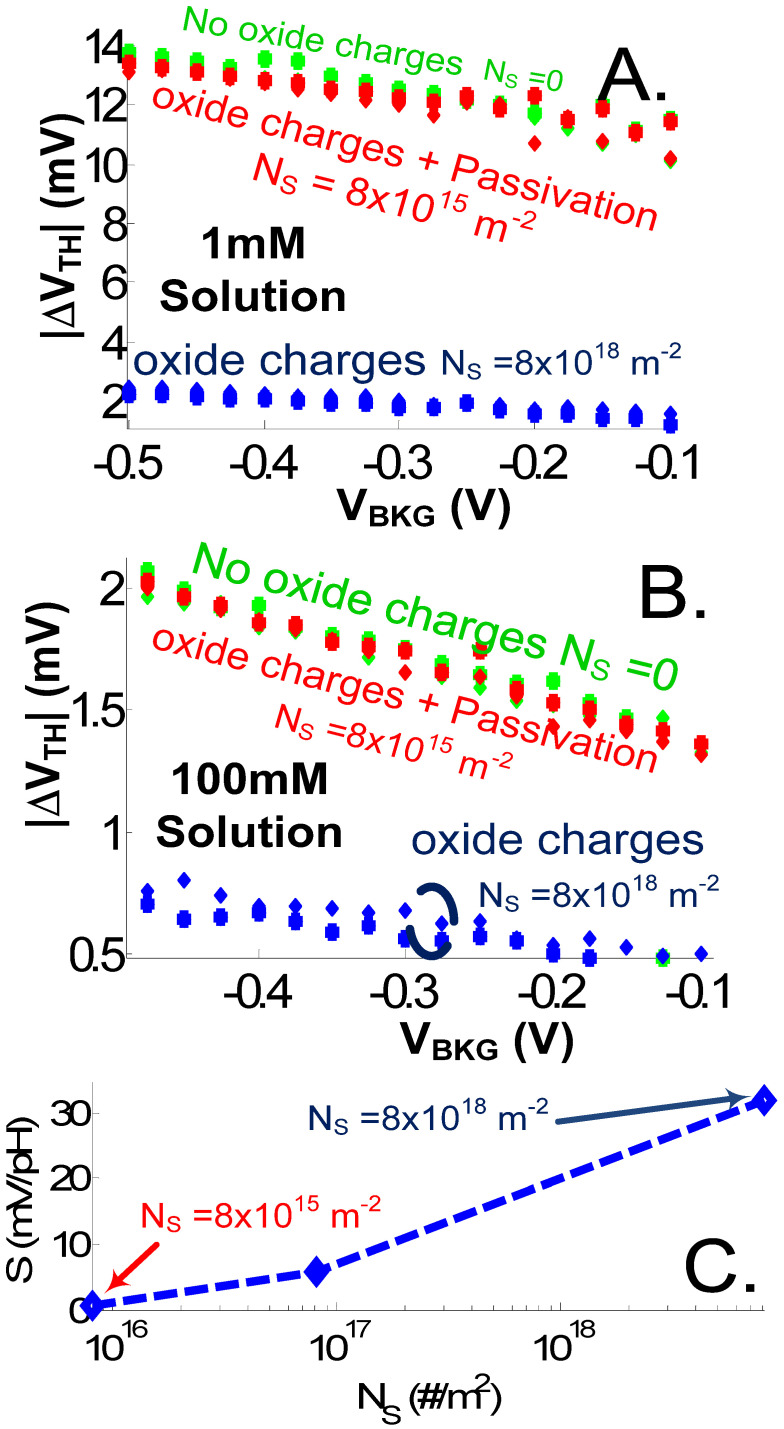
V_TH_ shift in function of V_BKG_ for a charged analyte with *z*_a_ = −1 and +1 without and with oxide charges for an Al_2_O_3_ oxide with different passivation (bare oxide (N_S_ = 8.10^18^ m^−2^) and alkyl-silane-passivated (N_S_ = 8.10^15^ m^−2^)) conditions in (**A**) 1 mM and (**B**) 100 mM solution. We fitted the alkyl-silane passivation effect on the site-binding model N_S_ parameter (Equation (2)) to the experimental NW pH sensitivity measurements of [32] before and after passivation. (**C**) Simulated impact of N_S_ on the NWT pH sensitivity (V_G_ = V_FG_, V_BKG_ = 0 V). d_SI_ = 3 nm. V_D_ = 0.7 V. L = 20 nm. Oxide: EOT = EOT_BOX_ = 0.5 nm. pH = 7, T = 300 K.

Figure 5A shows the strong reduction of S_+_ and S_−_ for the L = 20 nm 2 × 2 nm^2^ NWT case, when the surrounding media is changed from air to solution with c_ion0_ = 0, 1 (i.e., a typical buffered solution), and 100 mM (i.e., a typical undiluted physiological solution). As explained in [3], in solution, the higher media permittivity (ε_r_ = 80), when compared to air (ε_r_ = 1), causes the analyte-induced electric field to generate a wider but also much smoother energy barrier perturbation. S is further reduced for increasing ion concentrations due to screening. In solution, also, S quickly degrades with increasing charged-analyte-to-channel distances, e.g., when increasing the front-gate EOT or introducing a SAM layer (Figure 5B). Finally, as previously mentioned, H^+^ ions are responsible for the creation of a surface charge density at the oxide–solution interface (Equation (2)). Such a phenomenon is exploited, for instance, for pH sensing [24]. For biosensing, however, if the oxide is not passivated, *σ_0_* creates an additional screening effect that further reduces the sensitivity besides the direct screening of the ions (Figure 6A,B).

As shown here, these two screening effects, not the intrinsic sensitivity of the NWT itself, are the crucial effects to overcome to reach single-analyte detection in ionic solutions when using ultra-scaled NWTs. The reduction of the surface charge density is intrinsically linked to the used surface passivation technique [31]. In [32], experiments showed an important reduction of the NWT response to pH, down to the mV/pH range, after alkyl-silane functionalization. These results were found to be consistent, based on analytical models, with a reduction by three orders of magnitude of the density of available oxide sites N_S_ (Equation (2)) [32]. Our numerical simulations on the impact of N_S_ on the NWT pH sensitivity also confirm the same trend (Figure 6C). Furthermore, assuming such a passivation, our simulations show that in this case the original sensitivity (without oxide charge) can be restored (Figure 6A,B). Another solution may be the use of oxide-free NWBTs, providing a sufficient electrical and chemical insulation, and passivation can directly be provided by the SAM itself [2,6,31]. The sensitivity improvement measured in such devices is consistent with our theoretical calculations without oxide charges and a reduced analyte distance from the surface of the sensor [31].

Concerning ion screening, a publication has shown promising results by the application of a pulsed electrical signal that allows for recovering the unscreened sensitivity in transient regime with a time constant in the µS range [33]. Other publications have proposed to overcome the screening-induced performance limits of nanowire biosensors using electro-diffusion flow [34] or AC techniques in the MHz range [35].

### 3.2. Sensitivity and Limit of Detection in the Presence of a Single DNA Molecule

#### 3.2.1. Impact of Physical Parameters on the Sensitivity in Solution

We now investigate S of the NWT (assuming a well-passivated oxide) with or without (reference case) a 20-base-long DNA single strand attached on top of a 1nm SAM layer on the top gate, and using an uncharged PNA molecule as a selective probe (Figure 2). The device is biased through its fluid gate, while the back gate is grounded. For the DNA, we assume a non-permeable-to-ions, uniformly charged rod model with one charge per base [5,36]. The rod diameter is 1nm and each base has a length of 0.34 nm. The relative dielectric DNA permittivity is assumed to be 8 [37].

**Figure 7:**
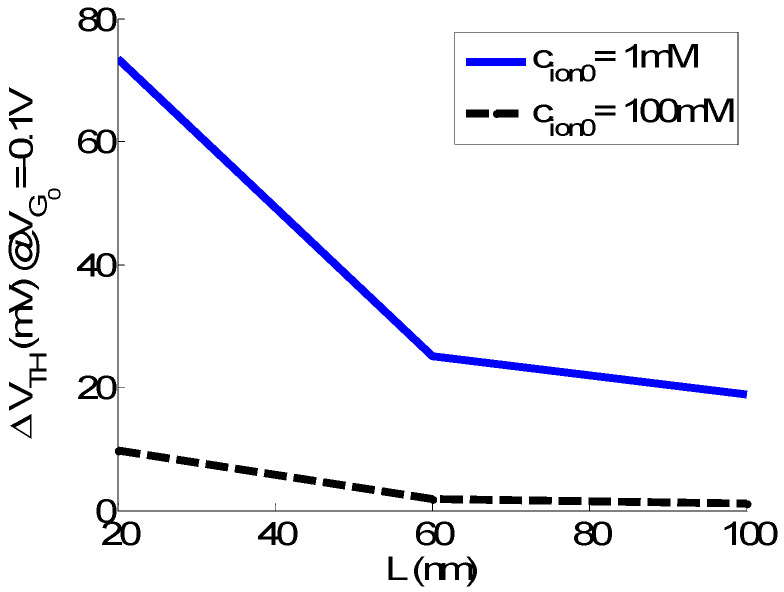
Simulated ΔV_TH_ (V_FG_) compared to the reference case for a 20B DNA analyte, extracted at V_FG0_ = −0.1 V in solution in function of L for c_ion0_ = 1 mM and 100 mM. V_D_ = 0.7 V. EOT = EOT_BOX_ = 0.5 nm. t_SAM_ = 1 nm. V_BKG_ = 0 V.

In Figure 7, the impact of L on ΔV_TH_ in solution is observed for two different ionic contents. S is enhanced compared to the single-charge case. This is due to, firstly, an increased number of charges to detect and, secondly, to the non-permeability to ions in the DNA case. As observed in the single-charge case, S decreases when increasing L. For the diluted solution (1 mM) case, however, the L = 100 nm NWT still presents a significant shift (ΔV_TH_ = 19 mV, that leads to about 30% of relative drain current variation σ_ID_). For the 100 mM solution case, on the other hand, only the L = 20 nm device seems to achieve a reasonable S for single DNA strand detection.

**Figure 8:**
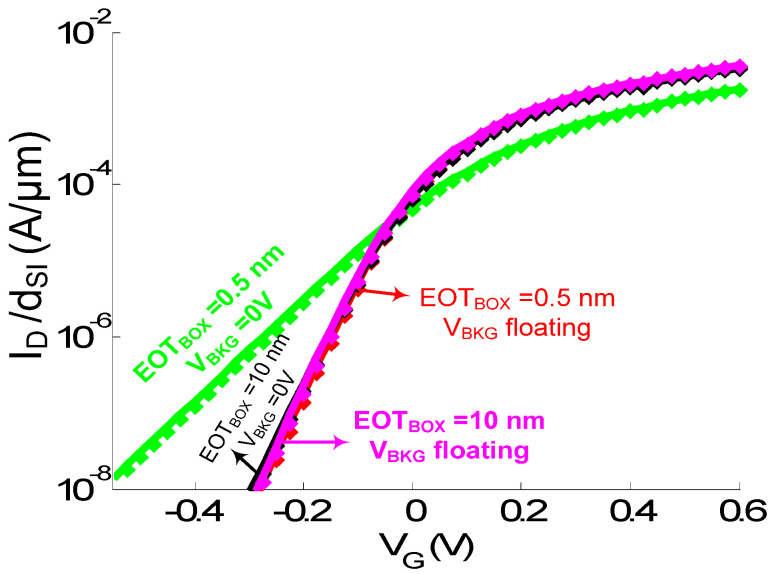
Simulated I_D_(V_G_ = V_FG_) curves of the NW with (symbols) and without (plain line) the DNA analyte in a 100 mM solution for two different EOT_BOX_ (=0.5 and 10 nm) and V_BKG_ (=0 V and floating) values. d_SI_ = 3 nm. L = 20 nm. V_D_ = 0.7 V. EOT = 0.5 nm. t_SAM_ = 1 nm. V_D_ = 0.7 V.

**Figure 9:**
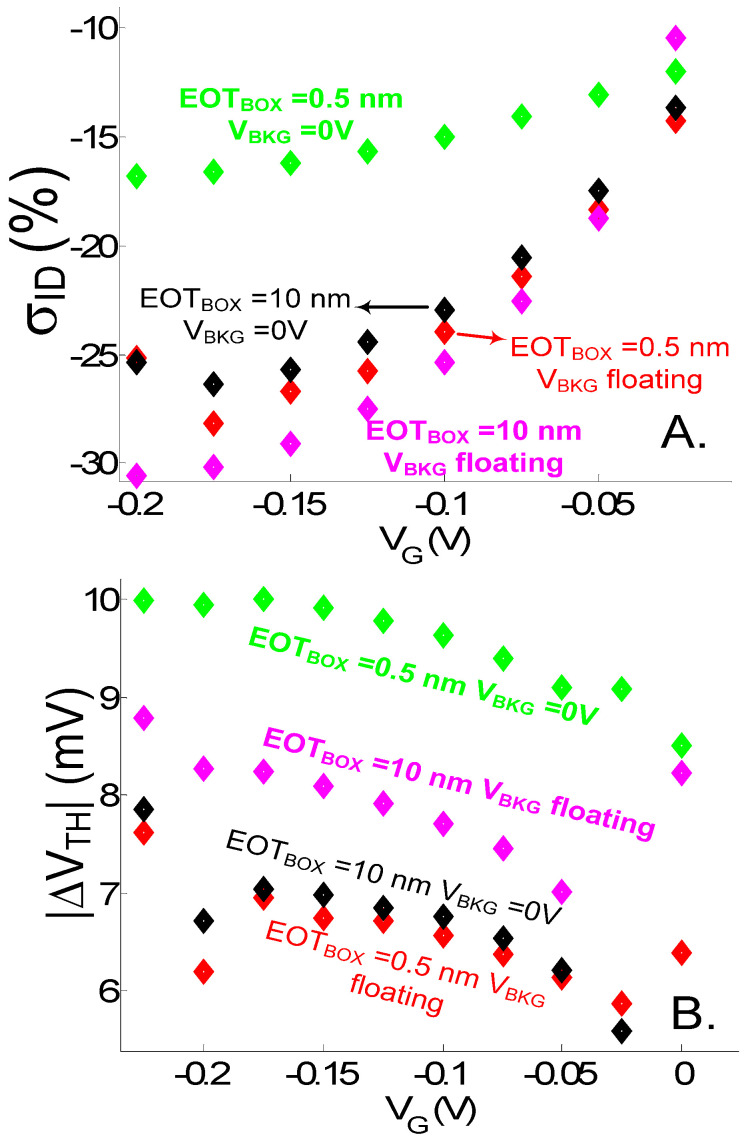
(**A**) Relative current variation σ_ID_ (%) and (**B**) V_TH_ shift in function of V_G_ = V_FG_ extracted from the I_D_(V_G_) curves of Figure 8. d_SI_ = 3 nm. L = 20 nm. V_D_ = 0.7 V. EOT = 0.5 nm. t_SAM_ = 1 nm. V_D_ = 0.7 V.

The impact of back-gate bias and EOT_BOX_ is investigated for the latter case (Figure 8 and Figure 9). When increasing EOT_BOX_ or letting the back gate float, σ_ID_ is increased (Figure 9A). A too-strong control of the back gate on the channel potential is indeed detrimental to the electrostatic shift (ΔE_C_) induced by the DNA molecule on the channel conduction band. However, the reduced front-gate coupling, induced when biasing the back gate (this can also be observed when looking at the reduced subthreshold slope in back-biased I_D_(V_G_) characteristics in Figure 8), amplifies the ΔV_TH_ shift related to ΔE_C_. Due to the reduced gate coupling, a bigger gate-voltage shift is needed to recover the same current level between charged and uncharged case. This explains why, despite achieving the lowest σ_ID_ variation (related to a lower electrostatic shift), the device with EOT_BOX_ = 0.5 nm and V_BKG_ = 0 V features the highest ΔV_TH_ shift (Figure 9B).

**Figure 10:**
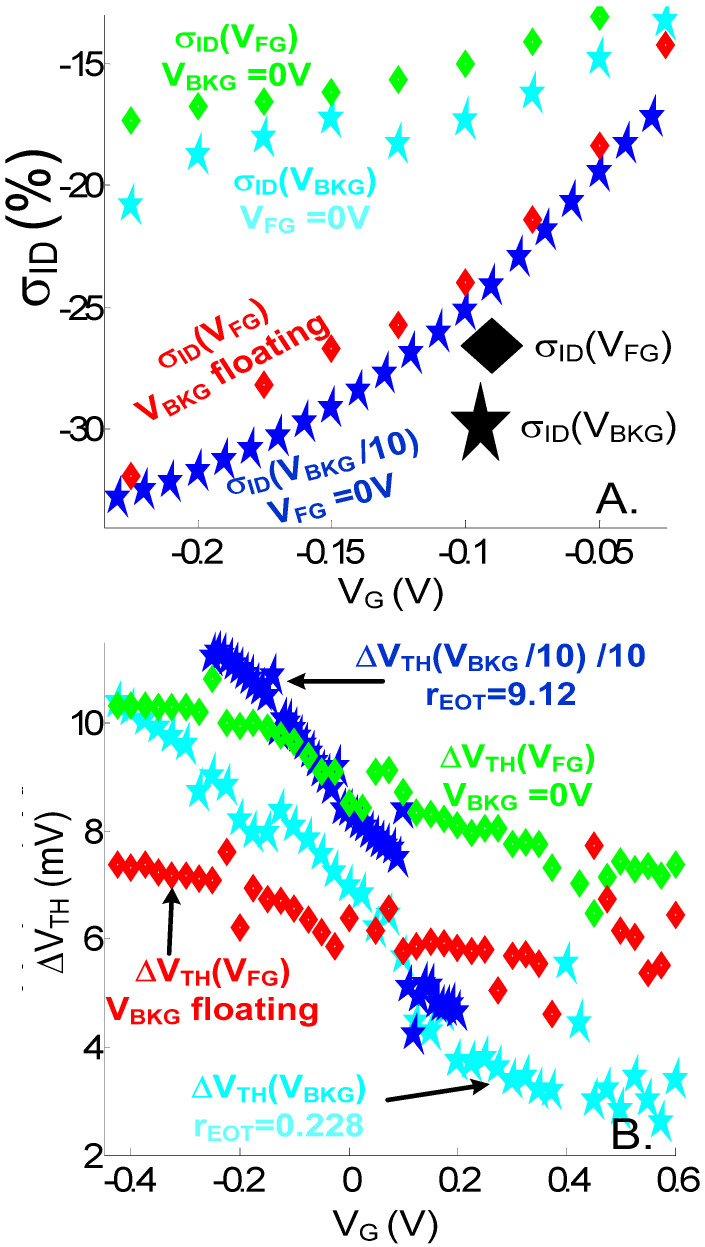
(**A**) Simulated relative current variation (%) and (**B**) V_TH_ shifts for front- (V_G_ = V_FG_) and back-gate (V_G_ = V_BKG_) bias for various capacitive coupling amplification factors r_EOT_ = EOT_BOX_/EOT_FG_. EOT_FG_ = 2.2 nm in all cases (EOT = 0.5nm. t_SAM_ = 1 nm (EOT_SAM_ = 1.7 nm)). EOT_BOX_ = 0.5nm for all cases except for the curves related to the NWT with back-gate bias and r_EOT_ = 9.12, for which EOT_BOX_ = 10 nm. As indicated in the legend for this device, the *x*-axis (V_G_) of its σ_ID_(V_BKG_) curve has been scaled (divided) by a factor 10, while both the *x*-axis and *y*-axis (ΔV_TH_) of its ΔV_TH_ (V_BKG_) curve have been scaled by a factor 10. d_SI_ = 3 nm. L = 20 nm. V_D_ = 0.7 V. c_ion0_ = 100 mM.

The relationships between back and front gate sensitivities both for σ_ID_ and ΔV_TH_ shifts feature similar considerations as just explained above (Figure 10). σ_ID_ being proportional to the electrostatic shift is not much changed, one exception being that it is not possible to enhance σ_ID_ by letting V_FG_ float. The I_D_(V_BKG_) curve at V_FG_ floating is in fact equal to that at V_FG_ = 0 V (assuming a 0 V work function for the ideal faradaic electrode in solution). This is due to the charge neutrality of the ionic solution being far from the nanowire that pins the solution potential for the floating case. ΔV_TH_ being further (inversely) dependent on the gate coupling is amplified by the front gate/back gate coupling ratio. As observed for pH sensors when back-gate-biased, ΔV_TH_ can therefore be amplified by the capacitive coupling amplification factor r_EOT_ = EOT_BOX_/EOT_FG_ [38,39]. EOT_FG_ is the total fluid gate EOT, which includes the oxide but also the eventual SAM dielectric layer (EOT_SAM_). This effect on ΔV_TH_ (V_BKG_) is well observed in Figure 10B for the device with EOT_BOX_ = 10 nm (r_EOT_ = 9.12), for which ΔV_TH_ in excess of 100 mV (the curve has been normalized by a factor 10, i.e., about equal to the r_EOT_ ratio) are achieved. For the device with EOT_BOX_ = 0.5 nm (r_EOT_ = 0.228), the reduced ΔV_TH_ (V_BKG_) is mostly observed in strong inversion regime. As observed in Figure 10B, ΔV_TH_ (V_BKG_) presents more variation with V_G_ and is typically bigger (resp. smaller) than ΔV_TH_ (V_FG_)*r_EOT_ in deep subthreshold (in strong inversion) regime. The stronger ΔV_TH_ (V_BKG_) dependency on V_G_ can be explained when taking into account the impact of the Si dark space regions related to quantum confinement in the computation of an effective r_EOT_ (r_EOT,eff_) > r_EOT_ (resp. < r_EOT_) [40]. r_EOT,eff_ decreases with V_BKG_ due to the migration of the channel centroid position toward the back interface when increasing V_BKG_.

#### 3.2.2. Limit of Detection

To resolve the conflicting or different impacts of back-gate coupling and back-gate biasing on ΔV_TH_ and σ_ID_ optimization observed in Figure 9 and Figure 10, one should optimize the limit of detection (LOD) and therefore consider how both sensitivity and noise signals are affected.

In a FET biosensor, the low-frequency noise (LFN), which originates either from the intrinsic 1/f LFN of the transistor itself or from the contact resistance LFN, has been shown to be dominating [39,41,42]. In an optimized Si technology, the 1/f LFN of the device is typically dominant and will be considered here [41]. Considering a number fluctuation noise, neglecting front-to-back-gate coupling effects but including correlated-mobility fluctuations [43], the current noise–power spectral density is given by [44,45]:

(9) $$S_{I}={\left(1+\alpha \mu_{0\;}c_{ox}\frac{I_{D}}{g_{m}}\right)}^{2}\times g_{m}^{2}S_{VFB}$$

(10) $$S_{VFB}=\frac{\delta K_{B}Tq^{2}N_{t}}{fWLC_{Ox}^{2}}$$

For silicon the tunneling attenuation distance, *δ*, and the Coulomb scattering coefficient, *α*, are typically equal to 0.1 nm and 10^4^ V·s/C, respectively. *N_t_* is the density of oxide traps, *WL* is the area under the gate and *C_ox_* the gate oxide capacitance per unit area. Other symbols have their usual meaning.

Integrating *S_I_* over the measurement bandwidth (between the low- and high-frequency cut-off *f*_1_ and *f*_2_, respectively), we can compute *δi_d_*, the total integrated noise, and then *SNR*, the signal-to-noise ratio:

(11) $$SNR=\frac{\Delta I_{D}}{\partial i_{d}}=\frac{\Delta l_{D}}{\sqrt{\ln\left(f_{2}/f_{1}\right)}\sqrt{S_{l}\left(f=1\;\mathrm{Hz}\right)}}$$

In Table 1, the equation above has been applied to compute the *SNR* from the NWT cases of Figure 10, taking Δ*I_D_* and *g_m_* from our NEGF simulations. We see that the front-gate-biased devices achieve a better *SNR*, one of the reasons being that the front-gate area is three times bigger that the back-gate area. The best case is achieved for the front-gate bias with grounded back-gate voltage (i.e., with maximum ΔV_TH_). This is because the equivalent gate-bias noise is about equal for both front-gate-bias cases (the noise is number-fluctuation dominated and we have neglected gate-coupling effects as a first approximation). As can be seen for the floating back-gate case, the higher current sensitivity is more than compensated by a higher current noise that leads to a lower *SNR*.

For the back-gate bias case with EOT_BOX_ = 10 nm, the device intrinsic *SNR* is also not improved, as the ΔV_TH_ amplification by about a factor of 10 is over-compensated by an increase of the noise. Our observations are also in line with those reported in [39], where despite an increased pH sensitivity, the intrinsic sensor device *SNR* was not improved.

## 4. Discussion

We have investigated, here, the fundamental charge detection limit of ultra-scaled NWTBs using a quantum microscopic NEGF approach. Our findings are that for negatively charged analytes, an N-doped NWT is more sensitive and should, therefore, be used. By analogy, a P-doped NWT is expected to be more sensitive to detect positively charged analytes. Our simulations predict S of tens to hundreds of mV/q in dry (air) or low-ionic solution environments, and single-charge/analyte detection seems within reach for a range of device dimensions (channel length, cross-sections) not too stringent compared to advanced CMOS fabrication capabilities. However, with typical ionic solution and SAM conditions, the sensitivity rapidly drops to the mV/q range. This translates in small I_D_ variations (a few % or less) and a narrow range of ultra-scaled device dimensions to reach single-charge detection. Finding ways to circumvent ionic screening (e.g., by using AC detection schemes) and oxide charges (e.g., by achieving proper passivation), as well as having the charge as electrically close as possible to the surface of the sensor, are crucial.

We have, then, extended our study to the detection of a single 20-base-long DNA molecule using a PNA-selective probe and investigated the impact of parameters such as back-gate biasing and BOX thicknesses to further enhance the sensitivity and the limit of detection. Our results seem promising for single DNA-molecule detection using ultra-scaled NWTs, in particular for the front-gate-biased, grounded-back-gate scheme, where a signal-to-noise ratio larger than 10 was predicted in a 100 nM solution. As for direct detection in physiological-like media, only an ultra-short nanowire seemed to yield sufficient sensitivity; Si NWTs could benefit from advanced nanoscale technologies and VLSI to be integrated in an array of single-analyte-sensitive and high-detection-probability DNA sensors. Again, to ease the trade-off between sensitivity and probability of detection, a detection scheme that overcomes ion screening is highly desirable.

## Figures and Tables

**Table 1:** Simulated NWT sensitivity and *SNR* for the different front- (V_G_ = V_FG_) and back-gate- (V_G_ = V_BKG_) biased NWT cases of Figure 10, at V_G_ = −0.2 V (−2 V for the case with EOT_BOX_ = 10 nm). Our NEGF simulations were used for ΔI_D_, whereas the noise was computed by integrating the noise spectrum in Equation (11). *N*_t_ was set to 2.3 × 10^18^ cm^−2^, which is typical for Si NWs [41]. The noise was integrated between *f*_1_ = 0.1 Hz and *f*_2_ = 5 Hz. If not specified otherwise, EOT_BOX_ = 0.5 nm. d_SI_ = 3 nm. L = 20 nm. V_D_ = 0.7 V. cion0 = 100 mM.

Biasing Scheme	|*σ*_ID_| [%]	ΔV_TH_ (mV)	*SNR*
V_G_ = V_FG_ V_BKG_ = 0 V	16	10	11
V_G_ = V_FG_ V_BKG_ floating	30	7.5	6.1
V_G_ = V_BKG_ EOT_BOX_ = 0.5nm	18	8.7	5
V_G_ = V_BKG_ EOT_BOX_ = 10 nm	32	118	3.1

## Data Availability

The data presented in this study are contained within the article and are available on reasonable request from the corresponding author.
